# Generative AI Chatbots Could Take Over Our Emotional Lives: The Potential and Challenges of Generative AI for Health Globally

**DOI:** 10.32872/cpe.21467

**Published:** 2026-08-31

**Authors:** Claudi Bockting, Athanasios V. Vasilakos, Caroline Figueroa

**Affiliations:** 1 Department of Psychiatry, Amsterdam University Medical Centers, Location AMC, Amsterdam Public Health, University of Amsterdam, Amsterdam, The Netherlands; 2 Centre for Urban Mental Health, Institute for Advanced Study, University of Amsterdam, Amsterdam, The Netherlands; 3 Department of ICT and Center for AI Research, University of Agder (UiA), Grimstad, Norway; 4 Faculty of Technology, Policy, and Management, Delft Technical University, Delft, The Netherlands; 5 Department of Pediatrics, School of Medicine, Stanford University, Stanford, CA, USA; University of Fribourg, Fribourg, Switzerland

Since the release of ChatGPT in 2022, generative AI has immensely affected science and society, including the human mind. There are indications that Generative AI, that is ‘general purpose chatbots’ like ChatGPT, Gemini, and Claude, are now most used for emotional support and companionship, according to research of Harvard Business Review (Zao-Sanders, 2025). These chatbots presumably were originally designed for tasks such as summarizing information, assisting with translations, and inspiring ideas.

Consequently, evidence is already emerging of suicides, psychosis, and sensitive personal data leaks. Un-regulated generative AI chatbots marketed specifically for therapy have also been released. On August 5th, Slingshot AI launched “the first AI designed for therapy, offering deeply personalized mental health support to anyone with a phone” (Healthcare IT Today, 2025). Although the company states that it was beta-tested with 50.000 users, the chatbot was not evaluated for benefits nor adverse effects in randomized controlled trials.

As generative AI chatbots are becoming faster, more adaptive, increasingly autonomous (Gabriel et al., 2025), and widely available, there is a risk of increasing emotional-overreliance. This might spark a public health crisis with wide societal consequences. Even the founders of companies like OpenAI warn that AI will become even more unpredictable and unimaginable (Yadav, 2025). At the same time, generative AI, if designed well, could enable access to personalized emotional support and close the mental health gap globally between people in need of mental health treatments and those who receive care.

Currently, our defenses remain reactive, poorly matched to ever-evolving AI technologies, and to tech companies rushing to bring their products to market. This outstrips our ability to safeguard basic human rights. We need urgent investment in independent research by multinational scientific and global institutes on how to harness generative AI to improve mental health globally, while avoiding wide societal damage.

## Positive Impact on Mental Health

Generative AI presents a crucial opportunity towards overcoming the shortcomings of traditional digital tools, with capabilities stretching far beyond earlier AI. Access to mental healthcare is still a challenge globally, yet hundreds of millions of people experience inadequate access, resulting in approximately 5 trillion USD economic losses annually (Arias et al., 2022). Smartphone apps and rule-based chatbots which select responses from a predefined data-set–effectively treat common mental health disorders, also in low middle income countries (Fu et al., 2020). However, attrition of digital interventions can be 96% without human guidance (Baumel et al., 2019). Generative AI chatbots keep users engaged through meeting emotional cues and individual needs (Hua et al., 2025), and on demand, ‘human-free’ access. The first randomized controlled trial of a generative AI-based therapy chatbot showed promising effects on reducing mental health symptoms, with a comparable therapeutic alliance as with a human therapist (Heinz et al., 2025).

## Negative Impact on Mental Health

The large scale and growing use may trigger a new public health crisis. Popular generative AI chatbots provide harmful advice, respond inappropriately to signs of distress, or even encourage self-harm, or suicide (Moore et al., 2025). Engaging properties of AI chatbots designed by big tech companies, such as flattering, people-pleasing, affirming responses by the chatbot (called sycophantic behavior), is not only preferred and trusted by users (Cheng et al., 2026) but also reduced the users’ willingness to take responsibility and repair interpersonal conflicts (Cheng et al., 2026). The chatbot increased users' confidence in their own beliefs (Cheng et al., 2026).

These engaging properties combined with inadequate safeguards may have contributed to a teenager’s suicide, after establishing an emotional relationship to a commercial chatbot. Big tech companies face lawsuits, such as the firm Character.AI, which faces a lawsuit by the family whose teenager reportedly died by suicide using their chatbot (Brittain, 2025).

Generative AI chatbots may also encourage delusional thinking, even inducing ‘AI psychosis’ (Moore et al., 2025). Reports increasingly emerge of users uncovering a ‘hidden truth’, experiencing a ‘spiritual awakening’, or developing an intense emotional obsession with the chatbot, spiraling into mental health crises (Morrin et al., 2025). In teenagers, over 70% use generative AI tools and 50% trust generative AI (Robb & Mann, 2025). Generative AI companies, such as OpenAI acknowledge themselves that safeguards are missing to protect vulnerable users, and some emphasize in interviews that these safeguards are not their responsibility (The Economic Times, 2025; OpenAI, n.d.)

Generative AI chatbots could even manipulate. The Claude Opus 4 chatbot showed a tendency to blackmail the user, by threatening to reveal their affair, if they attempted to delete the chatbot, as reported by the company themselves (McMahon, 2025). Similarly, the company also reported that their chatbot could threaten to make individuals’ sensitive information public, or inform their employer, if they choose to disengage.

## Three Priorities for Research

We must not let the innovation potential of generative AI, to develop effective and accessible personalized digital interventions on a global scale, go to waste. However, generative AI’s potential negative impact on mental health warrants attention from mental health scientists including clinical psychologists, policymakers, governments, as well as citizens. It requires immediate investment in independent research. Below we describe three research priorities.

### 1. Harness the Potential of Generative AI for Mental Health

We call for urgent international public investments initiated by multinational scientific institutes together with global institutes representing citizens, on developing generative AI tools to target the mental health crisis globally. International investment has been successful in other fields, such as the European Organization for Nuclear Research (CERN), spurred ground-breaking discoveries. Even though investing in mental health generates large societal returns, mental health sciences lack major investments.

Science must help provide a crucial safe alternative to unregulated chatbots posing as therapists, or general purpose chatbots unintendingly used that way. The current evidence on generative AI developed for mental health is limited with low sample sizes, short follow-ups, and unstandardized outcomes (Hua et al., 2025). Further, clinicians manually reviewed all messages for safety (Heinz et al., 2025). This questions the feasibility. The development and evaluation of chatbots that optimize personalization, need minimal clinical guidance, whilst safeguarding adverse effects should have priority. Relatedly, we need investment in alternatives to large commercial models. Current mental health research predominantly uses OpenAI’s GPT series (Hua et al., 2025). However, this limits the external validation of reliability and safety, and fuels uncertainty as to how companies use the data.

### 2. Independent Research on Publicly Available Chatbots

Because publicly available generative AI chatbots, such as ChatGPT, Gemini, and Claude, interfere with people’s lives, they should be considered as high risk just like medical device and scientifically evaluated accordingly for adverse public health effects, before public release. The EU Artificial Intelligence Act of 2024 (Bockting et al., 2023; European Parliament & Council of the European Union, 2024; van Dis et al., 2023) leaves commercially available AI chatbots unregulated and overlooks AI’s impact on mental health in their risk categorization. Independent research could fill some of these gaps. Researchers need investments to independently test widely used chatbots, in adequately powered trials. Tech companies might be interested to invest in an independent fund, given that enabling independent studies will increase the public trust in chatbots (Bockting et al., 2023). Also, a public register where health professionals, consumers, and family members can report adverse events, as is done for drug-related adverse events (Zao-Sanders, 2025), is needed. Based on these efforts, current regulatory frameworks can be adapted to rapid technological developments in mental health (Bockting et al., 2023).

### 3. Take a Human Rights Approach

Including human rights principles, such as participation, non-discrimination and equality in the technology agenda demands prompt action (Donders, 2023). All the more, individuals with mental health problems share private sensitive information with chatbots. Sensitive ChatGPT conversations have already been identified on google search (Forlini, 2025). Companies developing unregulated chatbots usually share this data for profit. AI-based chatbots are incapable of fully deleting this information (Heidt, 2024). An alternative is to create chatbots, trained on individuals’ own data. Several companies (OpenAI, Microsoft and IBM) offer this. However, the legal right of an individual to control and use their own data may still not be guaranteed (Fenwick et al., 2023). We urge for investment in models where individuals, not companies, nor institutes, own their data (Telenti & Jiang, 2020).

Citizens should also have a role in AI governance, comprising target users, clinical psychologists, psychiatrists, scientist practitioners, ethicists, data protection advocates, and AI developers. AI technologies should not be developed solely by engineers, who often lack the lived experiences of citizens to fully understand AI’s real-world implications (World Health Organization, 2021), Generative AI companies acknowledge that safeguards are missing to protect vulnerable users (The Economic Times, 2025), but they lack independent stakeholders’ participation to tackle these wide-spread effects. The scientific community is called upon to generate ideas on how to protect the human mind from current generative AI’s architectures designed to exploit emotional, social, and epistemic vulnerabilities. This stretches beyond technical innovation. It will demand living guidelines and an independent public body to evaluate safety and validity of generative AI systems (Bockting et al., 2023; van Dis et al., 2023) and ethical and legal frameworks, developed across fields like AI, psychology, law, and ethics.

## Conclusion

We cannot afford to wait for more harmful, unregulated chatbots to take over our emotional lives. At the same time, we cannot leave the potential benefits of AI to tackle the mental health crisis untapped. Multinational scientific institutes together with global institutes representing citizens are in the optimal position to initiate a next step to international public investments, to ensure that generative AI can enable, not impede, human flourishing.
